# Erratum

**Published:** 1799-04

**Authors:** 


We hope our readers will excuse the following errors, which escaped our attention in the first number, viz.

Page 13 to 17, for 'Dr. Dyer,' read *Dr. Dyce.*

